# Supplementary material for: Integrated Transcriptome and Metabolome Analysis of Rice Leaves Response to High Saline–Alkali Stress
Source: Int J Mol Sci. 2023 Feb 17;24(4):4062. doi: 10.3390/ijms24044062 (PMC9960601; doi:10.3390/ijms24044062)
Supplement: Supplementary file 1 [file ijms-24-04062-s001.zip › Supplementary material.pdf]

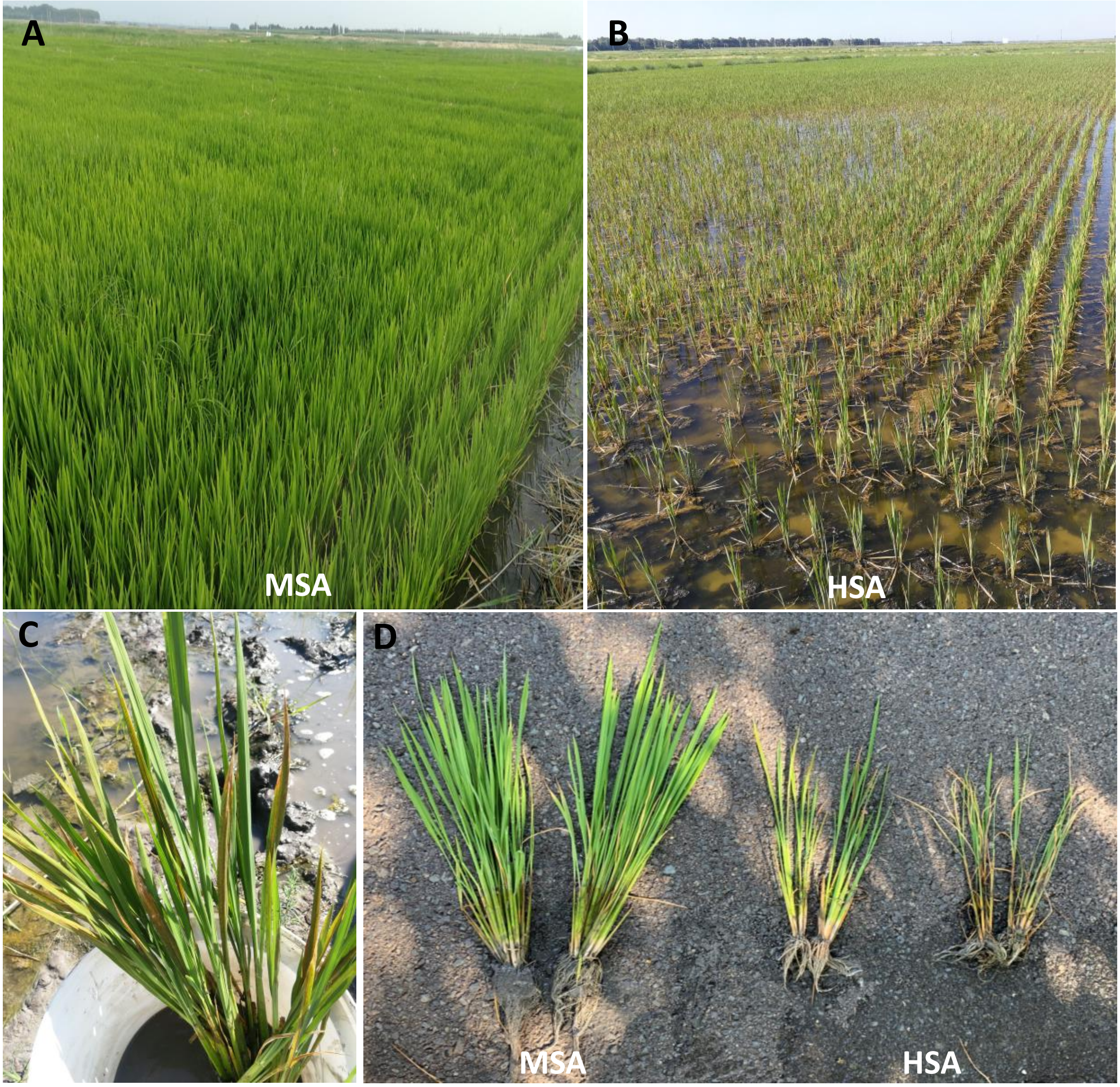

Figure S1. Field growth of rice at booting stage. (A, B) Rice grown in MSA (A) and HSA (B) paddy fields. MSA, mild saline-alkali. HSA, high saline-alkali. (C) Magnified image of rice leaves grown in HSA paddy field. (D) Comparison of rice plants grown in MSA and HSA paddy fields.

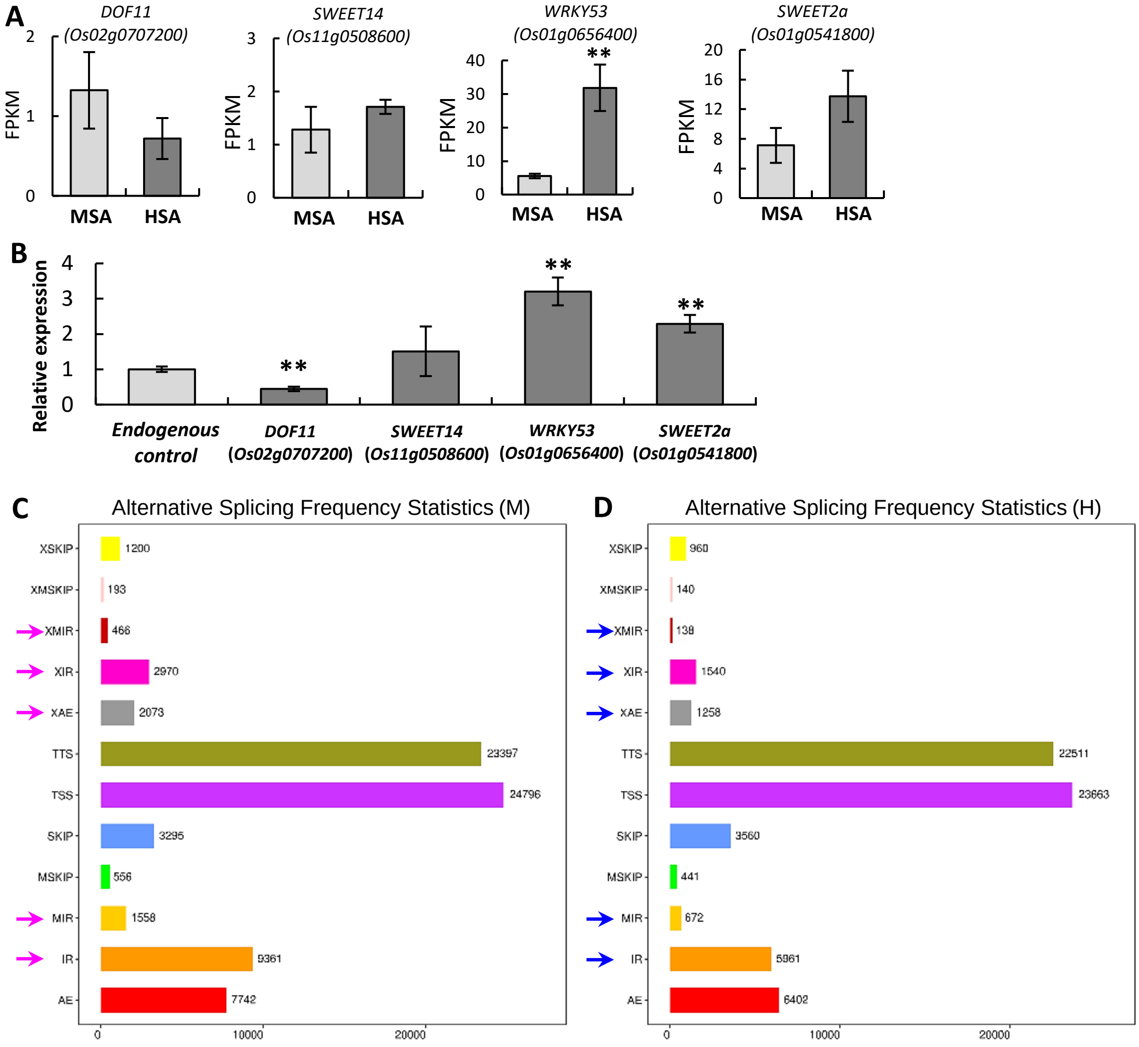

Figure S2. Differentially expressed transcription factors and alternative splicing in HSA and MSA groups. (A) Gene expression levels by Fragment per kilobase of exon model per million mapped reads (FPKM) value. (B) RT-qPCR determination of expression levels of the genes in A. (C, D) Statistics of alternative splicing frequencies of MSA and HSA groups, respectively. Arrows indicate the classes with significant differences between the two groups. Abbreviations: M, MSA; H, HSA.

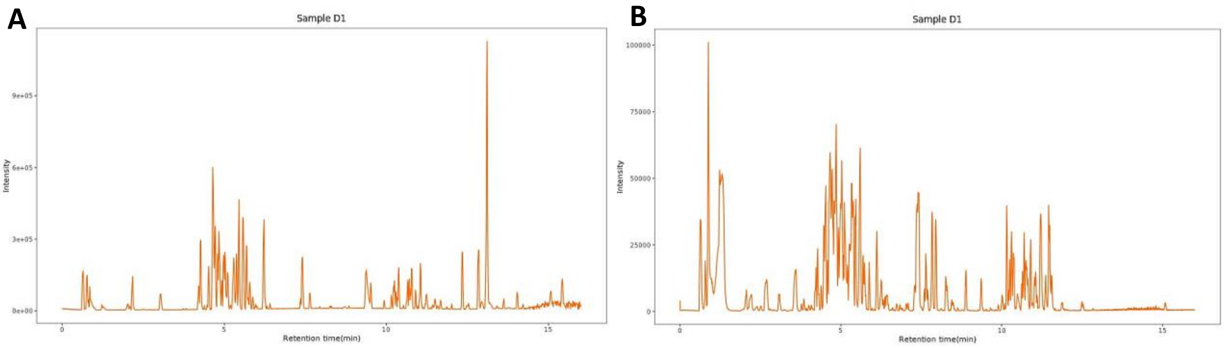

Figure S3. The total ion current (TIC) of the metabolites in positive ion mode (A) and negative ion mode (B).

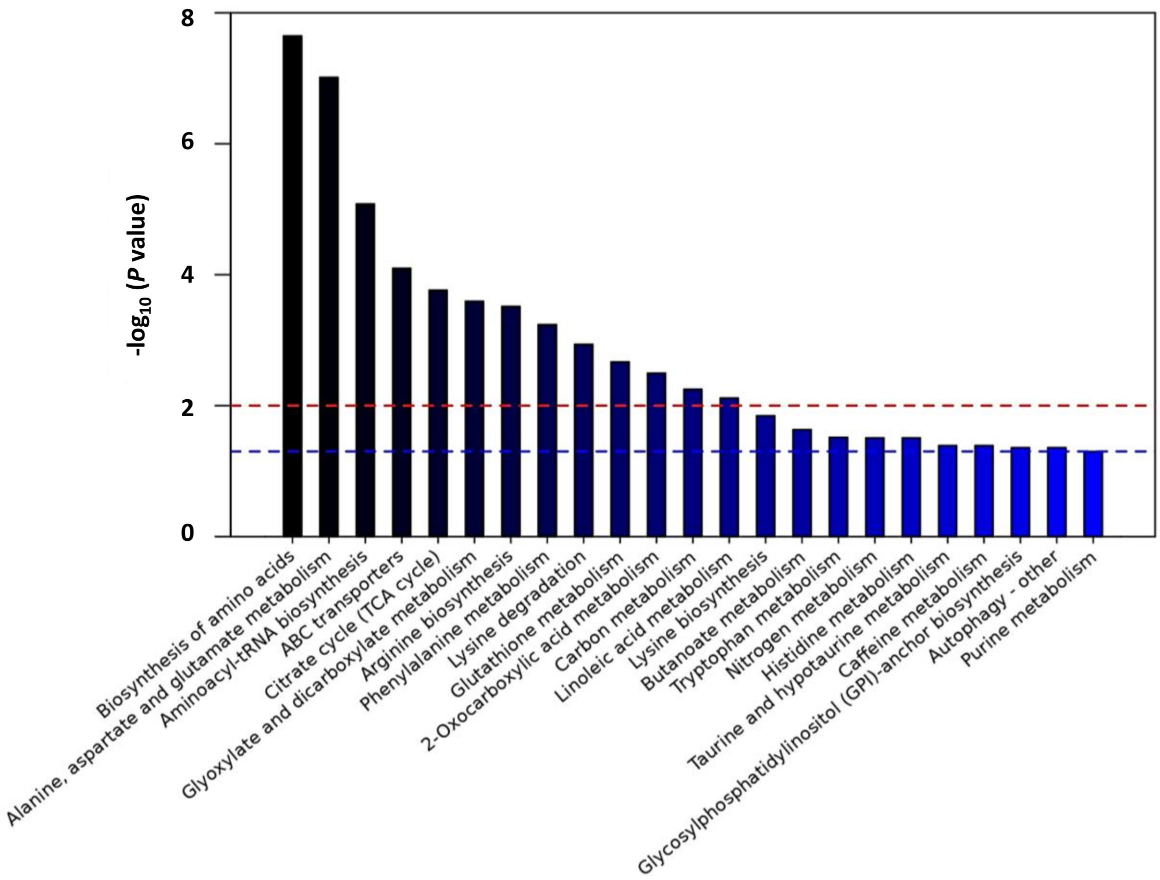

Figure S4. KEGG enrichment of differentially accumulated metabolites (DAMs). The DAMs were enriched in 23 metabolic pathways.

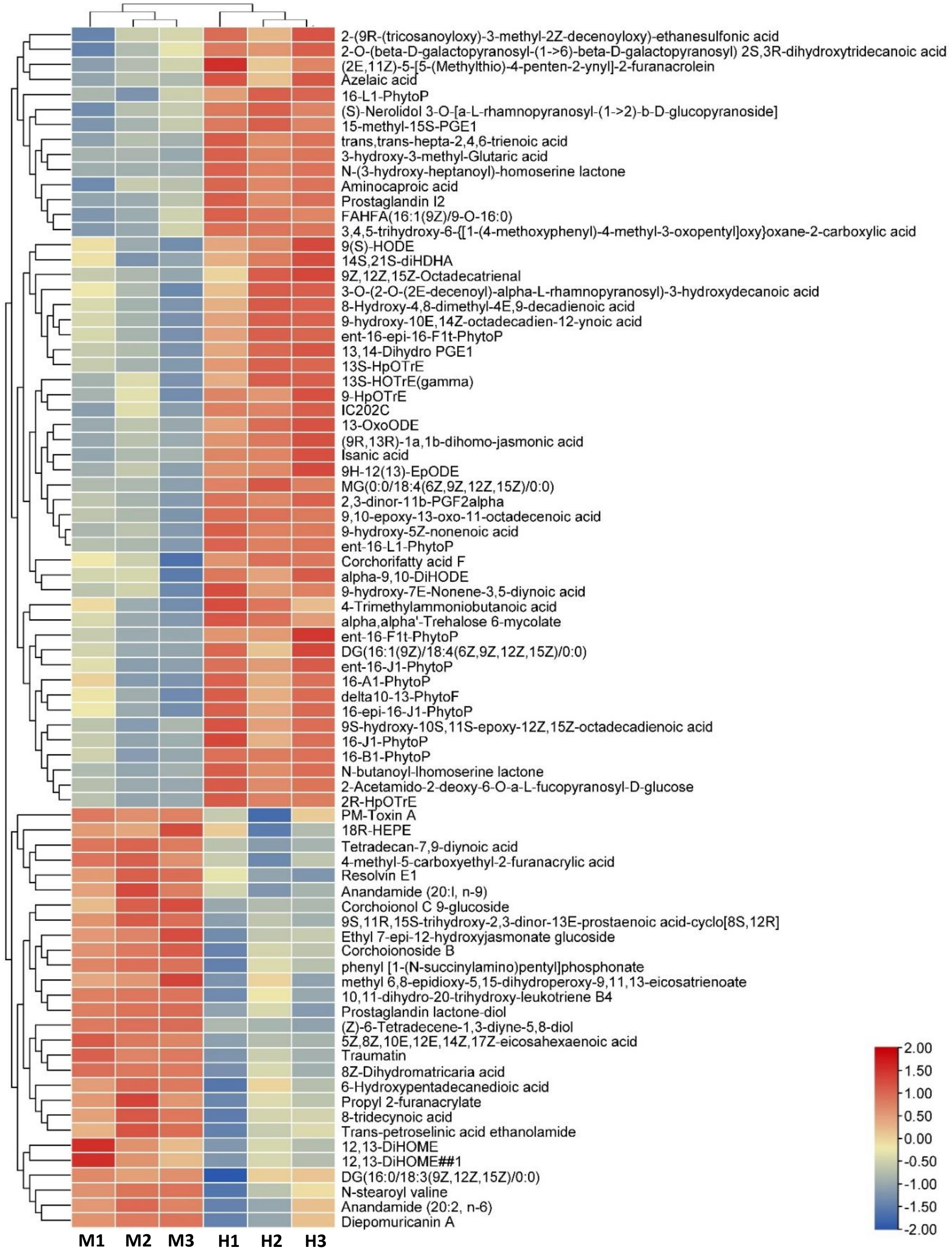

Figure S5. Clustering heat map of fatty acyls in DAMs. The alteration value of metabolites is normalized and shown in a color scale. Red, increase; blue, decrease. Abbreviations: M, MSA; H, HSA.

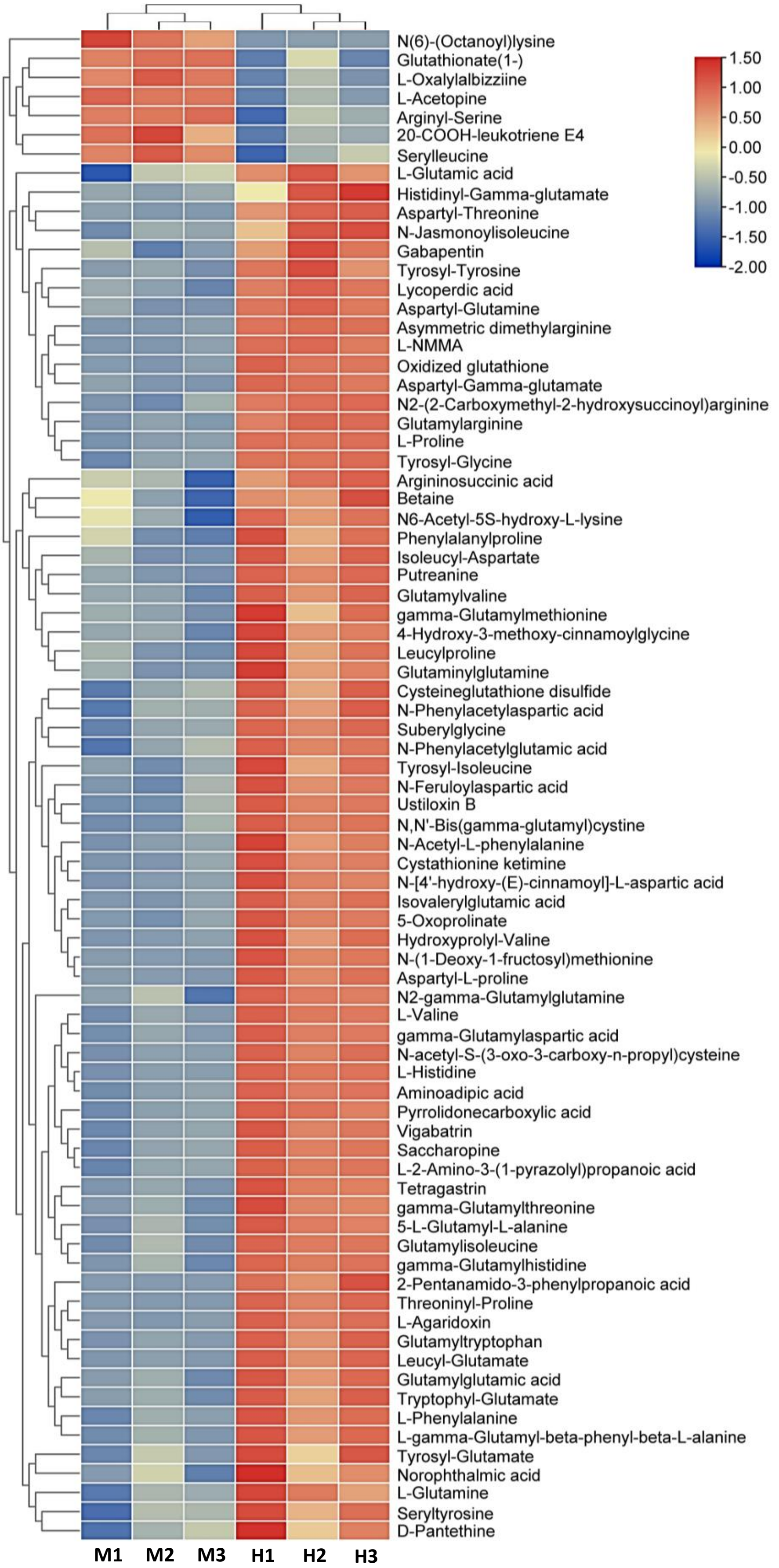

Figure S6. Clustering heat map of amino acids in DAMs. The alteration value of metabolites is normalized and shown in a color scale. Red, increase; blue, decrease. Abbreviations: M, MSA; H, HSA.

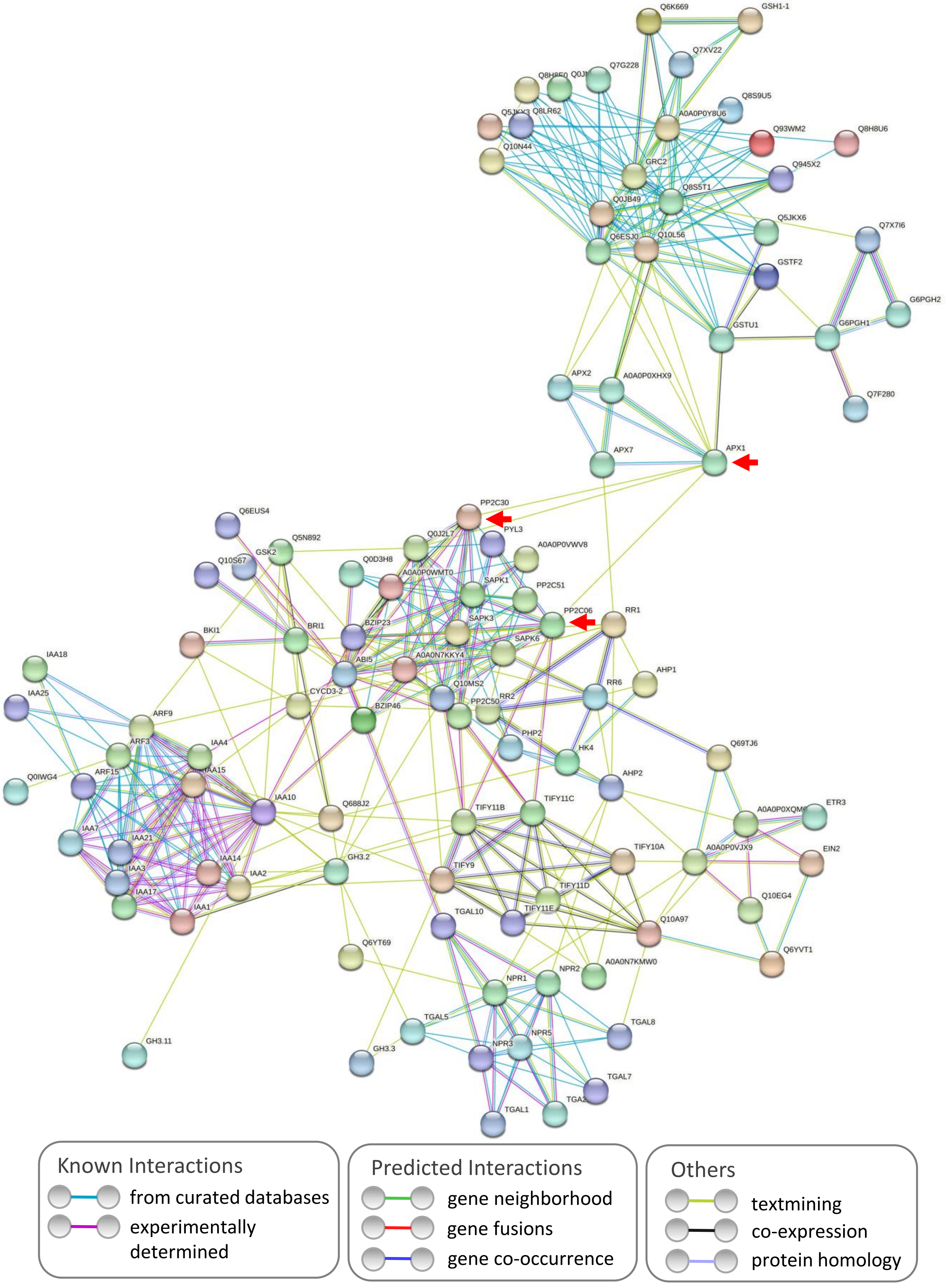

Figure S7. Prediction of protein-protein interaction network between Glutathione metabolic and Hormone signaling transduction pathways. The circles represent genes. The lines indicate interaction of two genes. The colors of the lines indicate the predicted information source annotated in lower panels.

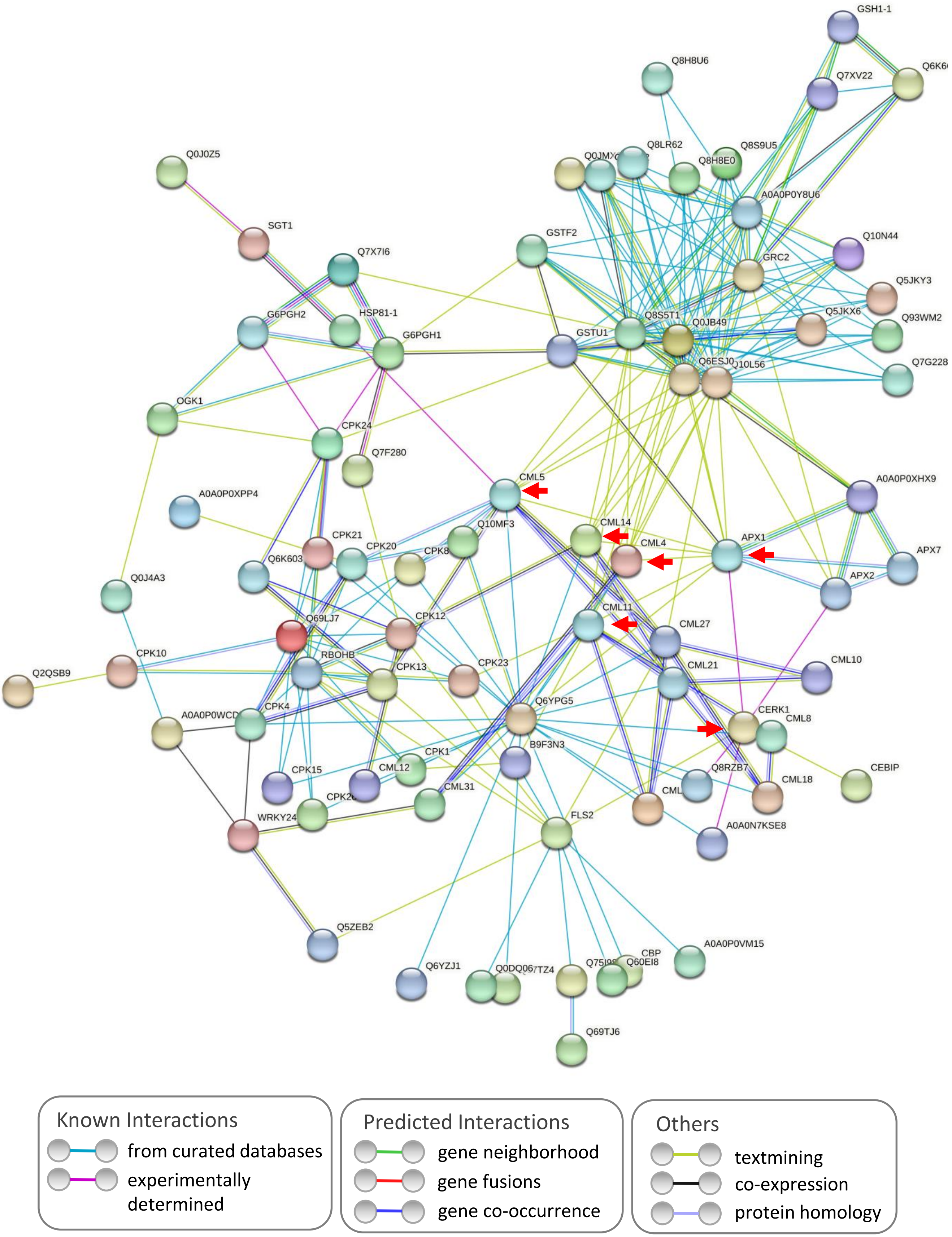

Figure S8. Prediction of protein-protein interaction network between Glutathione metabolic and Plant-pathogen interaction pathways. The meanings of circles, lines and colors are the same as those in Figure S7.
