# Supplementary material for: Integrated Transcriptome and Metabolome Analysis of Rice Leaves Response to High Saline–Alkali Stress
Source: Int J Mol Sci. 2023 Feb 17;24(4):4062. doi: 10.3390/ijms24044062 (PMC9960601; doi:10.3390/ijms24044062)
Supplement: Supplementary file 1 [file ijms-24-04062-s001.zip › Table S4.pdf]

Gene ID	F	R
Os02g0664000	GCTTCAAGGCCGAGTATCCA	AGTGGAGAAGTAGTTGGCGC
Os11g0484500	TCTGATCGTGGACAGGGAGT	TGTGTGCCCCAAACAAGTCT
Os10g0525800	GCATTTGTCAAGTGCAGCCA	CGGAACCTTGGTCTCGTTGA
Os12g0182200	AACGTCGATCTCAACTCCATTA	TTCTTGTAGAGTGCATCCAGAG
Os01g0151500	AGGATCGTCGACAACACCAC	GAAGAGGTGGTTCTCCGTGG
Os03g0337900	TGCTCAGGAGGTTGCTGAAG	TACAAACTTCTCGCCACGCT
Os04g0479200	TCTACTTCGAGACCTACGAGG	GAAGTTGGAGCAGTTGACGAG
Os02g0121800	AGTGCTACATTCTCTATCTCGC	GACCACATTTCTCTCTCAGACA
Os01g0654500	GATGAGTCAATCCGAGCTTTTG	CAGGATTGTGTTTTTGGTGCTA
Os10g0204400	AGGAACTAGAGAATTTTGGCGA	ATGTCATGTAATGCGTGTAACG
Os03g0700700	GATATCGAGAACCGGATCAAGG	AGCAATAGCACACGTATATCGA
Os03g0699700	CCTGTTGGAAAGTACCACTACA	GTCATTGTAAACATCGTACCGG
Os12g0559934	TGTTGCTCAAGGGCGAGG	TTTTCCGCGCGAACTCCT
Os04g0447100	GGCATCGATCGAAATTAATCAGG	CCCCAAACCAAAAGATACACTC
Os02g0707200	GTGCAAGAAGGAAAGGATTAGC	TACCATGCATGAGAAGATGGAA
Os11g0508600	GAGAAGAAGGTAGCTGCATGAG	TTTTTAGTCGTCGCACTCTCTC
Os01g0656400	GACTGCCAGATTAGCTACGAG	GGAGTACTTGTAGGTGTCCAAG
Os01g0541800	AGATGAACGCTCTCGGTTTAT	GCAGTTGAGTAGCGAGTAAATG
